# Environmental Assessment of Carbon Concrete Based on Life-Cycle Wide Climate, Material, Energy and Water Footprints

**DOI:** 10.3390/ma15144855

**Published:** 2022-07-12

**Authors:** Clemens Mostert, Jannik Bock, Husam Sameer, Stefan Bringezu

**Affiliations:** Center for Environmental Systems Research (CESR), Kassel Institute for Sustainability, University of Kassel, 34109 Kassel, Germany; jannik.bock@gmx.de (J.B.); husam.sameer@uni-kassel.de (H.S.); bringezu@uni-kassel.de (S.B.)

**Keywords:** concrete, carbon fibers, rebar steel, lightweight constructions, resource footprints, life cycle assessment

## Abstract

The construction industry contributes a major share to global warming and resource consumption. Steel-reinforced concrete (SC) is the world’s most important building material, with over 100 million cubic meters used per year in Germany. In order to achieve a resource-efficient and climate-friendly construction sector, innovative technologies and the substitution of materials are required. Carbon concrete (CC) is a composite material made of concrete and a reinforcement of carbon fibers. Due to the non-rusting and high-strength carbon reinforcement, a much longer life-time can be expected than with today’s designs. In addition, the tensile strength of carbon fibers is about six times higher than that of steel, so CC can be designed with a relatively lower concrete content, thus saving cement and aggregates. This research analyzes and compares SC with CC over its entire life-cycle with regard to its climate, material, energy, and water footprints. The assessment is done on material and building level. The results show that the production phase contributes majorly to the environmental impacts. The reinforcements made from rebar steel or carbon fibers make a significant contribution, in particular to the climate, energy, and water footprint. The material footprint is mainly determined by cement and aggregates production. The comparison on the building level, using a pedestrian bridge as an example, shows that the footprints of the CC bridge are lower compared to the SC bridge. The highest saving of 64% is in the material footprint. The water footprint is reduced by 46% and the energy and climate footprint by 26 to 27%. The production of carbon fibers makes a significant contribution of 37% to the climate footprint.

## 1. Introduction

With the current state and the future growth of the construction industry, ecological challenges arise, which are expected to be intensified in the future by the increased use of construction materials. The steadily growing world population, urbanization, and increasing infrastructure development are the major drivers of the global and continuously increasing demand for concrete and steel [1]. In addition to cement, natural aggregates, such as sand and gravel, are also required for concrete production. It is estimated that up to 50 billion tons of sand and gravel are extracted from natural ecosystems every year. As a result, this leads to land-use change, threats to biodiversity, and presumably to a future supply risk of sand and gravel [2]. In order to build infrastructures that can counteract environmental impacts, solution approaches such as resource-efficient technologies and the substitution of materials with more environmentally friendly alternatives are required [3]. The latter includes the physical replacement of conventional building materials with new, innovative ones, e.g., replacing cement with slag from steel mills [4]. The investigation of alternative materials, including increased use of calcined clay, engineered filler with dispersants, alkali-activated materials, or recycled aggregates is of ongoing interest to determine the contribution to reducing the related environmental impact [5,6,7].

Carbon concrete (CC) is a new type of construction material that is composed of two main components—concrete and a textile-like or bar-shaped reinforcement made of carbon fibers. The innovative composite material CC which has been investigated in various aspects since the mid-1990s has high-strength concrete matrices that result in a high load-bearing capacity in relation to the component thickness so that construction elements made of CC can be designed with a lower proportion of concrete compared to equivalent steel-reinforced concrete (SC) components [8]. However, high production costs and low demand are a disadvantage [9]. The reason for the high mass-specific price of carbon fibers is the cost-intensive manufacturing process of the precursor. The precursor is obtained from crude oil and forms the basic material for the manufacture of carbon fibers. Precursor manufacture accounts for over 50% of the manufacturing costs of carbon fibers [10]. Nevertheless, the carbon reinforcement is flexible and has a low self-weight, which enables filigree and curved concrete structures to be built [11]. Another advantage that emerges when using CC is the resulting weight savings compared to SC.

As a result, CC could turn out to be a resource-efficient option in the construction industry. With regard to the reduced use of materials, energy and water as well as the potential for the reduction of greenhouse gas (GHG) emissions, CC could emerge as a more environmentally friendly alternative than SC. However, there are only a few studies to date that have investigated this issue on the basis of a comprehensive environmental assessment [12,13,14].

This is the first study that examines and compares the environmental impacts of CC with SC based on resource and climate footprints. Life cycle assessment (LCA) in accordance with the European standard DIN EN ISO 14040 and 14044 is used to determine which composite material is more environmentally friendly [15,16]. The footprint assessment is carried out starting with an inventory on the material level for 1 m^3^ of concrete and then extended to the functional unit (FU) at the building level in order to be able to make a comparative assessment. Therefore, two equivalent bridge designs are investigated. An uncertainty analysis is also carried out at the building level. This study aims to strengthen the decision-making in favor of a more environmentally friendly material alternative in the structural design, planning and execution.

## 2. Materials and Methods

### 2.1. Life Cycle Assessment

An LCA study is a decision-making instrument for ecological product evaluations, comparisons and optimization [17]. The definition of the FU is a central aspect of the LCA to ensure the comparability of the examined products. Accordingly, the FU represents the basis for a neutral comparison of various options. A FU is a comparison unit that explains the central, quantified benefit or purpose of a product system [18]. In addition to quantitative measurements, qualitative aspects also play an important role in determining the FU.

The calculation of life-cycle footprints per FU has proven to be a suitable method for the ecological assessment of building materials [19]. A comparative footprint analysis was carried out for ultra-high-performance concrete compared to normal concrete. The environmental impacts were determined from cradle to grave, taking into account precast and ready-mixed concrete. The ultra-high-performance concrete shows higher environmental impacts per m^3^. When comparing different structural designs, it can be seen that a building structure with ultra-high-performance concrete has a lower footprint than with normal concrete.

This footprint analysis compares CC with SC. First, it starts with an inventory of process parameters and footprint data for 1 m^3^ of SC and CC. For the scalability of the results and comparison purposes, structure properties should be considered, which mainly affect the quantity of concrete in the end-use application. Second, the assessment is carried out on the building level, taking the performance and properties of the two building materials into account.

On the building level, two variants of a pedestrian bridge are compared, one made from CC and the other made from SC. Both variants connect two locations to overcome an obstacle or a cut in the terrain, have the same function and equivalent performance in terms of load capacity, bridge area, and traffic [20]. The FU is thus defined as the entire bridge considering a service life of 80 years.

### 2.2. Data

When determining the footprint data for 1 m^3^ CC and SC, it was taken into account that the material composition with regard to the mass- or volume-related share of concrete and reinforcement can vary between 1 and 5% depending on the application [21,22]. The concrete composition was taken from representative mix designs of similar types of concrete [6,23,24]. The CC consists of high-strength fine concrete C70/85 and the SC of C35/45 (Table 1).

1 m^3^ of CC consists of 0.985 m^3^ of special fine concrete with the compressive strength class C70/85 and 0.015 m^3^ or 27.01 kg of textile carbon reinforcement, which represent a percentage mass-related reinforcement of around 1.25%. For 1 m^3^ of SC 0.991 m^3^ of concrete with the compressive strength class C35/45 and 0.009 m^3^ or 69.16 kg of steel reinforcement are required.

For the comparison on the building level, a pedestrian bridge in Albstadt-Ebingen (Germany) is considered, which was designed with a bridge superstructure made of 6.4 m^3^ of CC [25]. A comparable design of the same bridge with a superstructure made of 16.5 m^3^ of SC is chosen [26]. The production of the bridge superstructures takes place in precast concrete plants including concreting, reinforcing, compacting and heating the reinforced concrete components. The fresh concrete in precast concrete plants is mostly poured into steel and wooden formwork (casting molds) [27].

The bridge was built in 2015, with a total length of 15.55 m, a width of 2.94 m and a load-bearing capacity of 4.66 kN/m^2^ [28]. The pavement slab is 90 mm thick. The top concrete layer of the pavement slab serves as a wear layer. This layer has a thickness of 10 mm to protect the structure from natural and mechanical stresses. The 14-ton bridge was produced as a complete prefabricated component [23,29]. The transport to the construction site was carried out by truck. The precast CC superstructure was then positioned and fixed on the abutments with two mobile cranes [26]. Finally, a steel railing was mounted on the trough walls and connected [28]. Thus, the bridges consist of two construction elements—a bridge superstructure made of CC and a railing made of steel.

### 2.3. System Boundary

**Figure 1:**
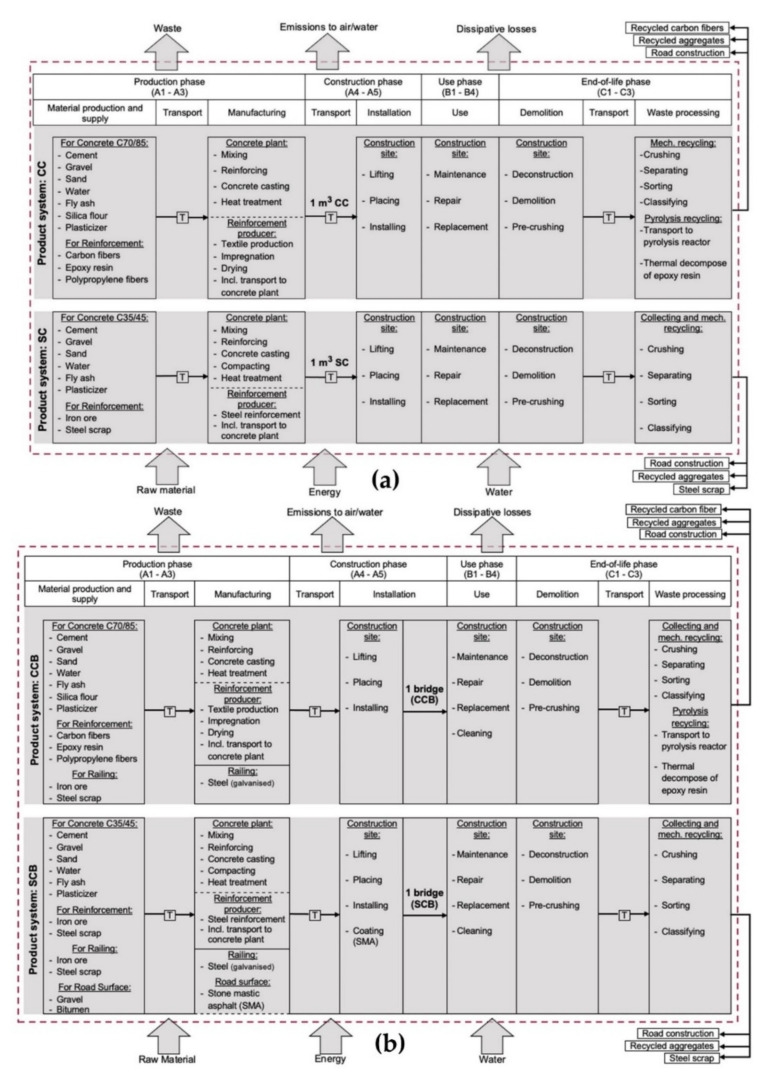
System boundaries of the life cycle assessment of (**a**) carbon concrete (CC), steel-reinforced concrete (SC) and (**b**) carbon concrete bridge (CCB), steel-reinforced concrete bridge (SCB) based on DIN EN 15804 [30].

The system boundaries are defined over the entire product life-cycle (a) for the materials and (b) for the compared bridge variants: carbon concrete bridge (CCB) and steel-reinforced concrete bridge (SCB). The material and bridge variants are assessed for the life-cycle modules A1 to C3 based on DIN EN 15804 [30] (Figure 1).

Raw materials, energy and water are taken from the natural environment and transferred into the technosphere. The processes result in waste, losses, and emissions which are released back into the natural environment. Modernization and improvement processes (module B5), as well as the use of energy and water for the operation of the technical building equipment (modules B6 and B7) during the usage phase were not considered. Module C4 describes waste disposal via landfill [30]. It is assumed, however, that, in accordance with the principles of circular economy and due to selective demolition, an optimal recycling rate is expected, so that neither concrete, steel nor carbon fiber waste fractions and other demolition materials are disposed of in a landfill. Accordingly, module C4 was not considered.

### 2.4. Footprints

The footprint assessment is performed for the climate, material, energy and water footprints. The climate footprint, also known as the carbon footprint, is calculated using the impact indicator GWI (global warming impact) expressed in kg CO_2_-eq. per FU based on the GWP values with a time horizon of 100 years (GWP_100_) as characterization factors (CFs). The relevant CFs are documented in the fifth assessment report (AR 5) of the Intergovernmental Panel on Climate Change [31].

The material footprint is calculated using two input-oriented impact indicators, the RMI (Raw material input) and the TMR (total material requirement) [32,33]. The RMI measures the cumulative amount of raw material, which is extracted from the natural environment and then transferred to the economic system to be further processed for the provision of products and services measured in kg raw material per FU. While the RMI only considers the raw materials (used extraction), the TMR takes into account the total primary materials extracted from the natural environment (used and unused extractions). As an impact indicator, the TMR measures the total amount of primary material that is required over the entire life-cycle measured in kg primary material per FU.

The energy footprint is determined by means of the input-oriented indicator CED (cumulative energy demand). According to VDI guideline 4600, the CED considers the primary energy-related demand that can be assigned to a product or service’s overall life-cycle phases [34]. As a result, the direct and indirect energy consumption along the entire product life-cycle is included. In the construction industry, the CED is an important indicator for the life-cycle wide analysis of building products [35]. A differentiation between non-renewable energy demand CED_non-renewable_ and renewable energy demand CED_renewable_ is recommended [36]. The use of fossil energy carriers causes a range of environmental impacts and can therefore be used to assess many different environmental problems. This makes the CED a good proxy indicator for the environmental performance of different materials [37]. In this assessment, the CED_non-renewable_ is applied calculated in MJ per FU.

To determine the water footprint, the AWARE Method (available water remaining) is used, which was developed by the WULCA (Water Use in Life Cycle Assessment) working group founded by the UNEP-SETAC Life Cycle Initiative [38]. This method considers not only the water consumption but also the regionally specific water availability. The amount of water remaining is calculated by the water availability in a country minus the water requirements for humans, plants, and animals to estimate the potential for water stress per FU [39]. 

The LCA modeling and footprint assessment were carried out in the software openLCA (version 1.10.3). Prefabricated data sets can be imported from different LCA databases, e.g., GaBi, ecoinvent or ELCD, to model product systems. The following GaBi databases (service pack 38) were used: GaBi Extension database XIV: Construction materials, GaBi Professional database 2019, GaBi Extension database VII: Plastics. The processes were predominantly chosen with the geographic reference of Germany (DE). Only for the manufacturing of the reinforcing steel via the basic oxygen furnace (BOF) route and for the manufacturing process of hot-dip galvanized steel for the bridge the geographic reference is Europe (EU).

### 2.5. Assumptions

When transporting the raw materials to the carbon fiber manufacturer, assumptions had to be made about the transport distances. Based on the assumption that there are fewer manufacturers of carbon reinforcement in Germany compared to other raw materials, e.g., cement and aggregates, longer transport distances are expected. It is assumed that the transport distances of the raw materials such as carbon and polypropylene fibers as well as epoxy resins to the reinforcement manufacturer are 5 to 10 times longer than the transport distances of the other raw materials to the precast concrete plants.

For the transport distance of the finished carbon reinforcement to the precast concrete plant, both at the material and building level, the distance between the reinforcement producer and the precast concrete plant is assumed to be 300 km.

In the case of the SCB, only the T-beam cross-section made of SC has static relevance. It is assumed that the railing is made of steel [26]. In comparison with the height, width, and length of the steel railing of the CCB with a weight of 0.83 t, a hot-dip galvanized steel railing with a weight of 1.5 t can apparently roughly be estimated for the SCB variant. In addition, a 2 to 3 cm thick top layer of stone mastic asphalt (SMA) is used as a road surface for the SCB to protect the C35/45 concrete. The estimation of the surface layer thickness and the installation amount of 30 to 50 kg per m^2^ to be used is derived from the “Additional Technical Contractual Conditions” (ZTV) for asphalt and SMA [40].

The different sizes result from the advantageous properties of CC. It is assumed that the general maintenance and servicing costs are lower than for SC due to the non-corrosive carbon reinforcement [41]. Based on Lünser [19], the expected service life of the materials and bridges is assumed to be 80 years.

When it comes to disposal, it is assumed that no waste collection or sorting losses occur. Only dissipative losses have to be taken into account with regard to the CCB. It is assumed that the 1 cm thick wear layer made of C70/85 concrete has been completely worn out due to mechanical abrasion and the effects of the weather during the use phase. These losses are released into the environment in an uncontrolled manner, so that this material cannot be reused or recycled.

For the mechanical processing of concrete, a delivery process is selected from the GaBi database, which considers the average processing of building waste in mobile and stationary processing plants. Based on the findings from Mostert et al. [42] an average transport distance of 50 km from the construction site to the recycling plant is assumed.

Another assumption must be made regarding the separation of the concrete fragments and the epoxy resin-soaked carbon fiber fragments. It is assumed that the separation by means of a camera-based single grain sorting is carried out according to type and without any sorting losses. However, for the camera-based separation process, which is carried out by a sensor-supported charge-coupled device color line camera [6], no cycle-specific power consumption could be determined. However, this electricity consumption is estimated to be very low, so it can be neglected in the LCA.

It is also assumed that the recycled aggregates, recycled carbon fibers or steel scrap generated from the treatment or recycling processes are then completely recycled or utilized in some other way. For example, recycled aggregates can be used for the production of recycled concrete [43].

## 3. Results

### 3.1. Footprints of Steel-Reinforced Concrete and Carbon Concrete

The footprints per 1 m^3^ of CC and SC are calculated by assigning the LCI results at the material level to the respective selected footprint categories. All results of the impact assessment with regard to the climate, material, energy and water footprints are related to the entire product life-cycle from A1 to C3 (Table 2).

The climate footprint of CC amounts to 1390 kg CO_2_-eq. per m^3^. The production phase (A1 to A3) has a significantly higher percentage contribution of over 80%. The percentage contribution from the usage phase is around 4.2% for the CC. This is due to the assumed lower maintenance of CC.

The end-of-life phase (C1 to C3) shows a noticeable deviation with 10%. Since the production phases contribute the largest share to the climate footprint, a more in-depth analysis is required, in which the percentages of individual materials or processes within the production phase (A1 to A3) are broken down and considered. This shows that cement production causes about 42% of the climate footprint of CC production. The production of the aggregates (sand and gravel) contributes 4% to the climate footprint of CC. The reinforcement in the CC, i.e., carbon fibers and epoxy resin, makes up almost 50% of the climate footprint.

The material footprint of CC is also mainly determined by the production phase. The aggregates represent more than 60% and the cement 29%. In the case of SC, well over 70% by aggregates and 21% by cement production.

The energy footprint of CC is 13,921 MJ per m^3^. The results of the energy footprint are also dominated by the production phase, with a share of 80%. The end-of-life phase of CC has significantly higher energy consumption. Analogous to the climate footprint, this is due to the high electricity and natural gas consumption for the pyrolysis of the epoxy resin-impregnated carbon fiber fragments. Around 72% of the energy footprint of the CC production is determined by carbon reinforcement production. This high proportion can be derived, among other things, from the upstream energy-intensive processes such as stabilization and carbonization for the production of high-tenacity carbon fibers. The main raw materials required for the concrete production of CC, such as cement and aggregates, have only a share of 18%.

**Figure 2:**
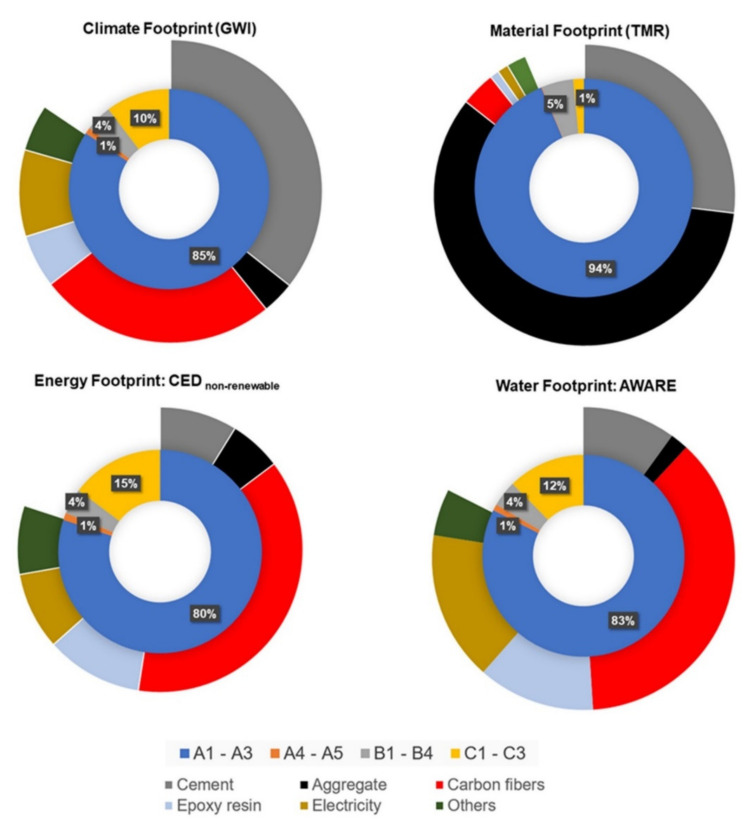
Share of climate, material, energy and water footprints of carbon concrete (CC), GWI: global warming impact, TMR: total material requirement, CED: cumulated energy demand, AWARE: available water remaining. A1-A3: production phase, A4-A5: construction phase, B1-B4: use phase, C1-C3: end-of-life phase.

The water footprint of CC is 9.92 m^3^ weighted water per m³. The production phase contributes to the water footprint with an equally significant share of over 80% mainly caused by the production of the carbon fiber reinforcement. In this regard, cement and aggregates production have a low share of around 14% (Figure 2).

### 3.2. Footprints of a Pedestrian Bridge Made from Carbon Concrete and Steel-Reinforced Concrete

**Figure 3:**
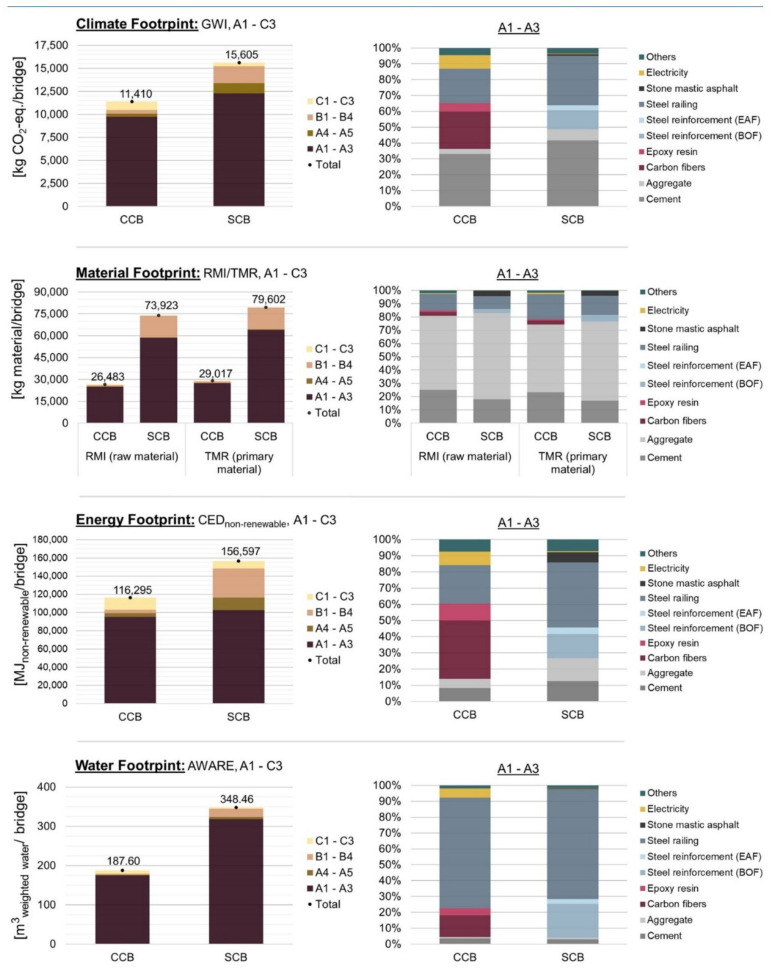
Climate, material, energy, and water footprints of a pedestrian bridge made from carbon concrete (CCB) and from reinforced concrete (SCB). A1-C3: entire life-cycle, A1-A3: production phase. GWI: global warming impact. RMI: raw material input. TMR: total material requirement. CED: cumulated energy demand. AWARE: available water remaining. EAF: electric arc furnace. BOF: basic oxygen furnace.

The results for the climate, material, energy and water footprints of the two bridge variants made from carbon concrete (CCB) and from steel-reinforced concrete (SCB) measured over the entire life-cycle are shown and compared (Figure 3).

The left side shows the absolute values of the footprint indicators, with a division of the four different product life phases (production, construction, use and disposal phase). In addition, the right side describes the percentage composition of the footprint indicators in relation to the production phase (A1 to A3) as the production phase shows the highest environmental impacts compared to the other life-cycle phases.

In terms of climate footprint, the CCB shows a total of 11,410 kg CO_2_-eq. compared to 15,605 kg CO_2_-eq. for SCB, corresponding to a saving of 27%. A reduction could be especially achieved in the production phase, which makes the largest contribution to the climate footprint compared to the other life-cycle phases. The reduction in GHG emissions is primarily due to lower production of materials for the CCB. The bridge superstructure is made from 6.4 m^3^ of concrete and 175.5 kg of carbon reinforcement. In comparison, more than 2.5 times the volume of concrete is required for the SCB superstructure and 6.5 times the mass of the reinforcement.

Further savings result in particular from the lower weight of the steel railing. In addition to the concrete and reinforcement, the production of the hot-dip galvanized steel railing contributes a significant percentage to the climate footprint in the manufacturing phase (22% for CCB and 31% for SCB). The materials for concrete production, such as cement and aggregates, contribute 36% to the CCB and 49% to SCB. The carbon reinforcement production (including electricity) causes more than 37% and the steel reinforcement production is around 15% of the climate footprint of the production phase.

In terms of material footprint, the CCB show even higher saving. With regard to the RMI and TMR values, the CCB requires 47,440 kg of raw material and 50,585 kg of primary materials less than the SCB, resulting in savings of well over 60%. One of the main reasons is the high savings in concrete, as significantly high amounts of cement, gravel, and sand are reduced, which have a relatively high impact on the material footprint. With more than 94% in the case of the CCB and around 80% in the case of the SCB, the production phase has the highest share of the material footprint. The large share of the use phase of the SCB can be explained by the high demand for maintenance and repair. The asphalt surface layer made of SMA had to be renewed three times during the assumed service life of 80 years.

The comparison of the energy footprints shows a lower primary energy consumption with regard to the CCB. With 116,295 MJ for the CCB, savings of up to 26% are achieved. Again, the production phase has the largest share of the energy footprint. However, the savings come on the one hand mainly from the lower energy demand for transportation and construction of the CCB superstructure (A4 to A5) and on the other hand from the lower demand for maintenance and repair in the use phase. For the CCB, for example, no asphalt top layer had to be removed, reconditioned or renewed several times. In the production phase, the energy footprint is dominated by the manufacture of carbon reinforcement (including electricity). This process contributes more than 54% to the CED_non-renewable_, compared to the hot-dip galvanized steel railing for the SCB.

The water footprint of the CCB shows a lower value than that of the SCB. The CCB requires 187.6 m^3^ weighted water and the SCB 348.46 m^3^ weighted water, resulting in savings of 46%. This saving can again be attributed to the production phase. The hot-dip galvanized steel railing shows a high contribution to the water footprint in the manufacturing phase of the CCB and the SCB. The high contributions of steel production can generally be explained by direct and indirect water consumption. Direct water consumption is mainly caused through steel production-specific processes such as cooling water. The indirect water consumption is mainly caused by the externally procured energy required for oxidation and iron ore reduction [44].

In summary, it can be seen that in view of the assessed footprints the CCB has 26 to 64% lower environmental impacts than the SCB over the entire product life-cycle. The highest savings could be achieved in the material footprint in particular. In the overall view, the substitution of infrastructures made from SC by CC could make a relevant contribution to climate protection as there is an urgency to reduce GHG emissions in order to achieve the 1.5 °C target of the Paris Agreement. 

Nevertheless, the reinforcement materials, especially the carbon fibers, contribute a not negligible high percentage to the climate, energy, and water footprint. By applying the footprint calculation to a practical example at the building level, the application-related advantages of CC were taken into account. The ecologically advantageous potential of CC results primarily from the high concrete savings.

### 3.3. Results of the Uncertainty Analysis at the Building Level

**Figure 4:**
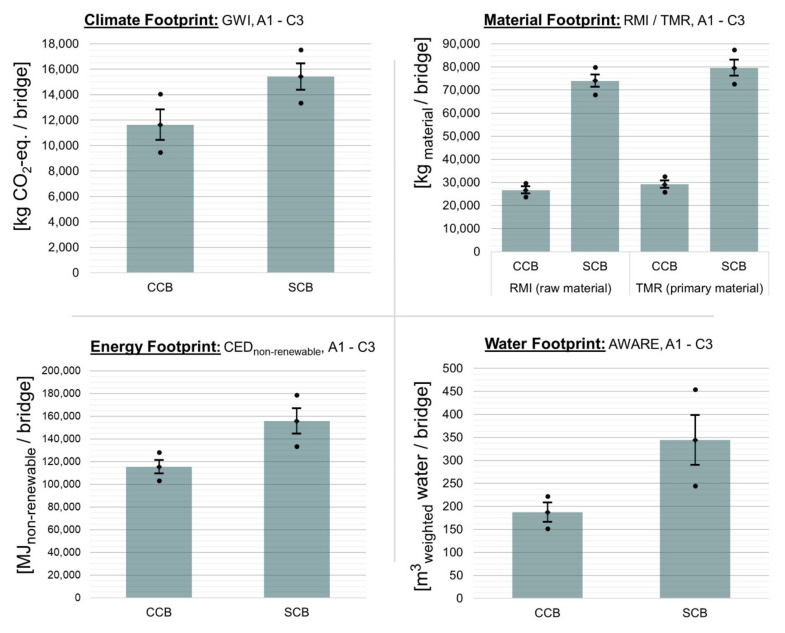
Uncertainties of the footprint results of a pedestrian bridge made from carbon concrete (CCB) and from reinforced concrete (SCB). A1-C3: entire life-cycle. GWI: global warming impact, RMI: raw material input, TMR: total material requirement, CED: cumulated energy demand, AWARE: available water remaining.

The results of the uncertainty analysis of the footprint results at the building level are shown in Figure 4. The mean value (column), the minimum and maximum value (lower and upper point), the 5%- and 95%-percentile (lower and upper antenna), and the median (diamond between the percentiles) are given. Accordingly, the percentiles define the 90% confidence interval.

With regard to the climate footprint, the results for the CCB are between 9462 and 14,070 kg CO_2_-eq. (coefficient of variation CV = 6.3%). In contrast, the results for the SCB are between 13,370 and 17,530 kg CO_2_-eq. (CV = 4.1%). In the worst case (minimum value SCB and maximum value CCB), the CCB could emit slightly higher GHG emissions than the SCB over the entire product life-cycle. However, given the confidence interval, this can be excluded with a probability of over 90%. In the best case (minimum value CCB and maximum value SCB), 8068 kg CO_2_-eq. and thus around 46% GHG emissions are saved.

The material footprint of the CCB is in the range from 23,800 to 29,700 kg of raw material and from 25,790 to 32,660 kg of primary material. The CV is 3.3 and 3.4%, respectively. With regard to the SCB, the values are between 67,980 and 79,900 kg of raw materials and between 72,690 and 87,350 kg of primary material. The CV is 2.2 and 2.6% respectively. It can therefore be assumed that significantly high quantities of 56 to 70% of raw materials and 55 to 71% of primary materials can be saved using the CCB. Primary fossil energy savings are in the range of 4 to 42%. This is due to the high uncertainty range of the SCB. The high difference between the minimum and maximum value can be justified by the assumptions regarding the weight of the steel railing (1500 kg ± 25%) with regard to the SMA top layer (1800 kg ± 25%) and production routes (BOF and EAF) of the steel reinforcement.

The water footprint of the CCB is in the range between 152 and 222 m^3^ weighted water (CV = 6.8%) and in the range between 245 and 454 m^3^ weighted water (CV = 9.6%) for the SCB. This means that savings on water footprint are in the range of 9 and 67%. The overall assessment shows that the standard deviations of the material, energy, and water footprint with regard to the CCB are lower than those of the SCB product system. This fact is due, among other things, to the high absolute deviation and the great influence of the weight of the hot-dip galvanized steel railing. Apart from the climate footprint, there is a 100% probability that environmental impacts can be reduced by manufacturing the bridge from CC. It is certain that significantly large quantities of raw and primary materials will be saved, especially through the use of CC for the bridge superstructure of the CCB.

## 4. Discussion

The footprint results per m^3^ do not take into account the different material properties and performance capabilities. Nevertheless, the footprints on the material level provide the basis for further calculations of the resulting environmental impacts on the building level. The data can be used in an application-related LCA of entire components and structures. It is important to include the different capacities at the material level, for example, by comparing the amount of SC and CC used with the corresponding load-bearing capacity.

It should also be mentioned that the results only relate to prefabricated SC or CC components or structures. A holistic ecological assessment of CC and SC for reinforcement or renovation measures with ready-mixed concrete was not carried out. Their environmental impacts can differ in comparison to the environmental impacts of precast concrete components. Further research is needed in this regard.

The main reason why the CCB has lower environmental impacts than the SCB in each footprint category is that it requires significantly less concrete and thus a lower overall resource input. The high tensile strength and corrosion resistance of carbon fibers also allow for a more flexible design of components in freeform geometries. Taking into account the entire component life-cycle and an extended service time, the benefits of CCB in terms of reduced environmental impacts would increase even further. However, despite intensive research on the material properties, the long-term durability of CC is not yet fully understood [45].

A major limitation in the study results is the fact that the substructure of the two bridge variants was not included in the LCA. This would probably lead to even more favorable results for the CCB because smaller abutments are required due to the lower bridge weight. In order to minimize the limitations of the study, further research, expert interviews, and surveys of companies are required.

Another limitation is related to the time reference of data, especially in assessing long-lasting infrastructures. The considered data for the LCI represent the status quo. The current state of the art was taken into account for the production, construction, use, and end-of-life phase for the respective product systems. However, it can be expected that future technological development will change production and, in particular, disposal processes in favor of greater resource and energy efficiency. In principle, an increase in the assumed service life would lead to lower footprint results.

As the cut-off approach is applied for LCA modeling, no environmental credits are given for the secondary materials obtained from waste management. According to the current state of the art, it is expected that the original quality of the carbon fibers is reduced after recycling [45,46,47]. As a result, the recycled carbon fibers can only be reused in what is known as downcycling [13]. In contrast, the steel reinforcement can be recycled without any major loss of quality [48]. These aspects could be taken into account if other LCA approaches are applied [13].

As mentioned above, there are only a few studies to date that have compared CC with SC based on a comprehensive environmental assessment [12,13,14]. The general results of the footprint assessment, especially in relation to climate and energy footprint, corroborated the findings of these studies. First, the materials and processes required for the carbon reinforcement have a significant influence on the environmental impacts of CC. Second, within the product life-cycle, the production phase has the largest contribution to the environmental impacts. Third, in contrast to the results at the material level, the results of the assessment on the building level show that structures made from CC could have lower environmental impacts in all footprint categories.

The application of different end-of-life scenarios can lead to different results [49]. In the footprint assessment, the waste management of CC has a significantly higher environmental impact than that of SC, since the pyrolysis process of the carbon fiber fragments is energy intensive [13].

The material footprint, quantified by the input-oriented impact indicators RMI and TMR, as well as the water footprint, using the AWARE method, were not examined in any of these studies. Significant new findings could be achieved in this regard. The assessment shows that the CCB consumes significantly less raw materials and water than the SCB mainly due to the high material savings.

## 5. Conclusions

The innovative building and composite material CC has a high potential to be a more environmentally friendly material than SC, because of its performance and material properties. Due to the corrosion-resistant carbon reinforcement with a very high tensile strength, components and structures made of CC require significantly less concrete. In the analyzed case study, around 60% less concrete had to be used for the CCB compared to the SCB. However, it should be taken into account that as long as there are no standards for the use of CC regarding load-bearing structural elements, the savings cannot be achieved in building practice.

The ecological assessment of CC and SC based on the climate, material, energy, and water footprints was the primary goal of this research. For this purpose, CC and SC were analyzed on the one hand at the material level and on the other hand at the building level. Based on DIN EN 15978, the entire product life-cycle was taken into account [50]. Accordingly, the production, construction, use, and end-of-life phases were assessed using the openLCA software and the GaBi database. Furthermore, uncertainties were quantified using Monte Carlo Simulation.

At the material level, comparing 1 m^3^ of CC and SC, it was found that CC, apart from the material footprint, has significantly higher environmental impacts than SC. The material footprints of the two building materials are in the same range. Except for the water footprint, the results for CC show higher uncertainties than for SC. However, in order to take into account the performance and the favorable material properties of CC, a LCA was carried out at the building level, in which two functionally equivalent bridge variants were examined. The results show that the bridge variant made of CC has lower environmental impacts than the bridge variant made of SC in each footprint category:

- Material footprint—64%;
- Climate footprint—27%;
- Energy footprint—26%;
- Water footprint—46%.

Nevertheless, the results of the SCB are subject to higher uncertainties with regard to the material, energy and water footprint. The relative savings would increase considerably if a longer service life was assumed for CC. It was shown that infrastructures made from CC can save considerably large amounts of concrete due to its advantageous properties. Despite their low mass-related proportion, the reinforcement materials make a significant contribution to the environmental impacts. In particular, the carbon fibers used in CC and the steel used from the BOF production route in steel-reinforced concrete, along with cement, make a relatively high contribution to the climate, energy, and water footprints. The material footprint is largely determined by the aggregates (sand and gravel) and the cement, and less by the raw materials for the reinforcements.

However, it is not only raw materials that can be saved but also transport and logistics costs, especially if precast concrete components are applied [51,52]. CC could thus represent a material- and cost-efficient alternative in the construction industry. While the production of carbon fibers is still largely based on petroleum, it could be also manufactured by carbon sequestered from the atmosphere [53].

The common use of CC requires more studies in the future. Due to the relatively high production costs of CC, it is also sensible to analyze and evaluate CC from an economic point of view to determine the life-cycle costs. An eco-efficiency analysis is necessary so that the decision-makers receive a comprehensive assessment of CC. If infrastructures made of CC are proven to be economically advantageous over the entire service life compared to SC, this could be an important factor for the widespread use of CC. In addition, the assessment and analysis of social aspects along the complete supply chain of CC and SC should play a more important role in the future.

A more detailed assessment with regard to carbon fiber production is required. The comparison of the conventional carbon fiber production from crude oil and the innovative new production processes using carbon, capture, and utilization (CCU) technologies could therefore be of great interest. More research work will be needed in the field of carbon fibers recycling, especially with regard to high-quality recycling.

## Figures and Tables

**Table 1:** Mix design of carbon concrete (C70/85) and steel-reinforced concrete (C35/45).

Material	Unit	1 m^3^ Concrete	
		C70/85	C35/45
Cement	kg/m^3^	500	356
Aggregates	kg/m^3^	1700	1808
-Gravel	kg/m^3^	1105	796
-Sand	kg/m^3^	595	635
-Crushed stones	kg/m^3^	-	377
Water	kg/m^3^	183.4	165
Fly ash	kg/m^3^	60	47
Silica fume	kg/m^3^	35	-
Additives	kg/m^3^	15	1.8
**Total**	kg/m^3^	2493.4	2377.8

**Table 2:** Resource and climate footprints of steel-reinforced concrete and carbon concrete per 1 m³ for the life-cycle phases (A1-A3), (A4-A5), (B1-B4) and (C1-C3); for carbon concrete compressive strength class is C70/85 and mass of carbon fibers is 27.01 kg; for steel-reinforced concrete compressive strength class is C35/45 and mass of steel reinforcement is 69.16 kg.

Category	Climate Footprint	Material Footprint	Material Footprint	Energy Footprint	Water Footprint
Indicator	GWI	RMI	TMR	CED_non-renewable_	AWARE
Unit	kg CO_2_-eq./m^3^	kg_raw material_/m^3^	kg_primary material_/m^3^	MJ_non-renewable_/m^3^	m^3^_weighted water_/m^3^
steel-reinforced concrete	611	3530	3640	4409	7.00
carbon concrete	1390	3591	3657	13,921	9.92

## Data Availability

Not applicable.
